# Effects of Gushukang for postmenopausal osteoporosis: A protocol for systematic review and meta-analysis

**DOI:** 10.1097/MD.0000000000020908

**Published:** 2020-07-02

**Authors:** Guangwei Wang, Liwei Huo, Guocai Chen, Huayong He

**Affiliations:** a Guangzhou Orthopedic Hospital; b The Fifth Clinical Medical School, Guangzhou University of Chinese Medicine, Guangzhou, China.

**Keywords:** Gushukang, meta-analysis, postmenopausal osteoporosis, protocol, systematic review

## Abstract

**Background:**

Postmenopausal osteoporosis (PMO) is one of the most common systemic bone diseases with a high risk of fracture. Traditional herbal formula Gushukang (GSK) has been used to treat PMO. However, there is no systematic review related to GSK for PMO. The object of this work is to evaluate the efficacy and safety of GSK in the management of PMO.

**Methods:**

We will search the PubMed, Embase, MEDLINE, Cochrane Library Central Register of Controlled Trials, China national knowledge infrastructure database (CNKI), Wan fang database, Chongqing VIP information, and SinoMed from their inception to May 2020. All randomized controlled trials (RCTs) of GSK for the treatment of PMO will be included. The improvement of vertebral fracture and bone mineral density (BMD) will be accepted as the primary outcomes. The meta-analyses will be performed by using the RevMan 5.3.

**Results:**

This study will provide a high-quality comprehensive evaluation of the efficacy and safety of GSK for treating patients with PMO.

**Conclusion:**

The conclusion of our systematic review will provide evidence to judge whether GSK is an effective intervention for patients with PMO.

**Trial registration number:**

10.17605/OSF.IO/MKN3F.

## 1 Introduction

Postmenopausal osteoporosis, one of the primary types of osteoporosis, is a metabolic bone disorder with attributes of low bone density that leads to bone fragility.^[1]^ It is a worldwide health issue with serious consequences and a heavy financial burden.^[2]^ According to a nationwide study in China, the average prevalence of osteoporosis in Chinese women aged 50 years and over was 44.1%.^[3]^ The most important adverse health effect of PMO is the occurrence of bone fractures, which affected the health-related quality of life of women.^[4]^ Moreover, osteoporotic fractures are usually associated with high morbidity and mortality.^[5]^ Therefore, with the aging Chinese population, early and effective management of PMO is of great significance to improve the quality of life and reduce the rate of disability.

In recent years, more and more attention has been drawn to the treatment of PMO with traditional Chinese medicine (TCM). As a vital part of complementary and alternative medicine, TCM has been used to treat PMO for a long time with its multi-target and fewer side effects.^[6]^ According to the characteristics of TCM-defined syndromes, kidney deficiency was the major pathogenesis of PMO.^[7]^ Kidney deficiency can reduce the level of estrogen-regulated by the gonadal axis, thereby causing osteoporosis, and kidney-tonifying Chinese medicine can relieve osteoporosis by increasing estrogen levels.^[8]^ Gushukang (GSK), a marketed prescription in China (approved No. Z20060270), was clinically applied to treat PMO. Clinical study has demonstrated that GSK could prevent and treat osteoporosis effectively through increasing estrogen and androgen.^[9]^ Besides, experimental researches have shown that GSK exerts beneficial effects on trabecular bone by regulating vitamin D and calcium metabolism, and exhibits protective effects on promoting bone formation and preventing osteocyte apoptosis through the BMP-2/Smads signaling pathway.^[10,11]^ Although the therapeutic effects of GSK on PMO were confirmed by many studies, there is still a lack of high-quality evidence to support the effectiveness and safety of GSK on patients with PMO. In this work, we will perform a systematic review to evaluate the efficacy and safety of GSK in the treatment of PMO to provide evidence-based guidance for clinical application.

## 2 Material and methods

This study will be performed following the Preferred Reporting Items for Systematic Reviews and Meta-analysis (PRISMA) Statement.^[12]^ We have registered this work at Open Science Framework (OSF, https://osf.io/). The registration DOI of this study is10.17605/OSF.IO/MKN3F.

### 2.1 Inclusion criteria

#### 2.1.1 Type of studies

In this work, randomized controlled trials (RCTs) that explore the specific efficacy and safety of the GSK in the treatment of PMO will be included. Non-randomized control studies and observational study will be excluded.

#### 2.1.2 Types of patients

All patients with a confirmed diagnosis with postmenopausal osteoporosis will be included. There will be no restrictions on ethnicity, economic status, severity of the disease, or education.

#### 2.1.3 Types of interventions and comparisons

Patients in the treatment group must have been treated using GSK or combined with routine treatment recommended by guidelines, and the control group must have no treatment, received placebo or routine treatment alone. If the treatment group and control group have received routine treatment, the routine treatment must be consistent. Any researches including other Chinese patent medicine, Chinese herb, and acupuncture will be excluded. The treatment durations should be at least 6 months.

#### 2.1.4 Types of outcomes

The primary outcomes will focus on the improvement of vertebral fracture and bone mineral density (BMD). The secondary outcomes included the visual analog pain (VAS) score, estradiol (E2), urinary calcium creatinine ratio, serum calcium, serum phosphorus, bone gla protein (BGP), alkaline phosphatase (BALP), quality of life, and adverse events.

### 2.2 Search strategy

This study search will be mainly based on electronic databases, including PubMed, Embase, MEDLINE, Cochrane Library Central Register of Controlled Trials, China national knowledge infrastructure database (CNKI), Wan fang database, Chongqing VIP information, and SinoMed. We will search these databases from their inception to May 2020. We will also search Google scholar, Baidu Scholar to find out other related literature. Two authors (GW and LH) will search and screen all the citations independently. A search strategy that combines MeSH terms and free words will be adopted. The search strategy was as follows:

1#: Search ((gushukang[MeSH Terms]) AND gushukang granule[Title/Abstract]) AND gushukang keli[Title/Abstract].
2#: Search ((((osteoporosis[MeSH Terms]) AND Osteoporosis, Postmenopausal[MeSH Terms]) AND primary osteoporosis[Title/Abstract]) AND postmenopausal osteoporosis [Title/Abstract]) AND postmenopausal [Title/Abstract].
3#: Search ((((((((randomized controlled trial[Title/Abstract]) AND RCT[Title/Abstract]) AND controlled clinical trial[Title/Abstract]) AND randomized[Title/Abstract]) AND randomly[Title/Abstract]) AND random[Title/Abstract]) AND controlled[Title/Abstract]) AND control[Title/Abstract]) AND trial[Title/Abstract].
#1 and #2 and #3

### 2.3 Study selection and data extraction

#### 2.3.1 Selection of studies

**Figure 1:**
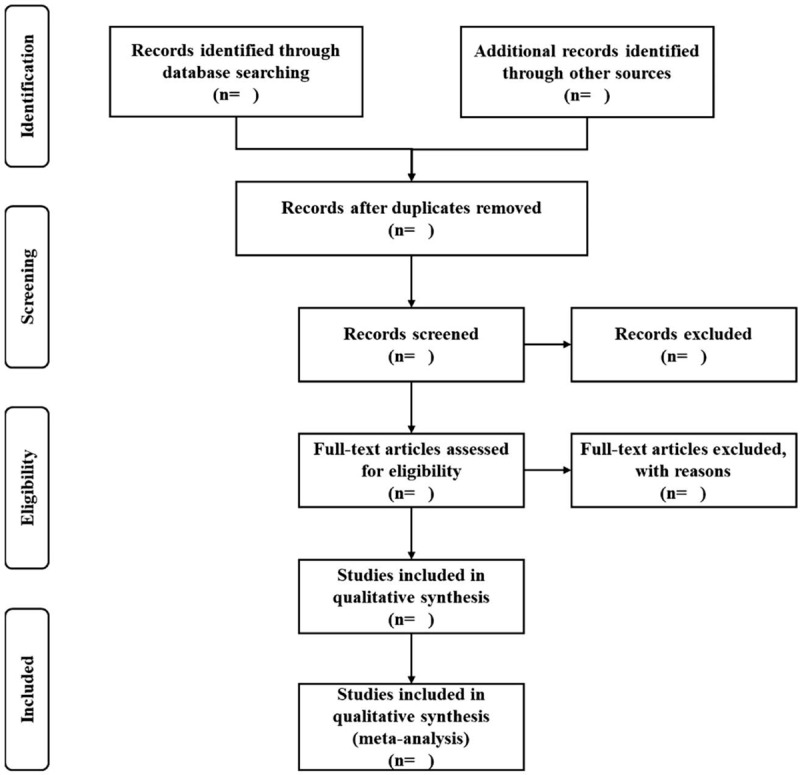
Flow chart of study selection.

Based on the research criteria and search strategies, 2 reviewers (GW and LH) will review the topics and abstracts independently. The eligible articles will be further accessed for inclusion by reading the full version. Any different opinions will be resolved through discussion with other reviewers. The details of the selection process are shown in Fig. 1.

#### 2.3.2 Data extraction and management

Firstly, the results of these data extraction will be checked. Then, we will extract and record the first author's name, year of publication, study design, intervention, sample size, duration of intervention, and outcomes. If there is not enough data, we will contact the corresponding author for more detailed information.

#### 2.3.3 Risk of bias assessment

Two reviewers will assess the risk of bias by using the Cochrane collaboration tool independently. The methodological quality of the RCTs will be evaluated through 7 items, including random sequence generation, allocation concealment, blinding of participants and personnel, blinding of outcome assessment, incomplete outcome data, selective reporting, and other sources of bias. These studies will be evaluated as “Low risk,” “High risk,” or “Unclear risk.” Any disagreements will be solved by a discussion of all reviewers.

#### 2.3.4 Measures of treatment effect

For dichotomous data, the risk ratio (RR) with 95% CIs will be used. For continuous outcomes, the mean difference (MD) or standard MD (SMD) with 95% CIs will be utilized for analysis.

#### 2.3.5 Assessment of heterogeneity

Statistical heterogeneity will be calculated by Cochrane *X*^*2*^ and *I*^*2*^ tests.^[13]^ If *P* ≥ .05 and *I*^2^ ≤ 50%, it suggests that there is no statistical heterogeneity or the heterogeneity is small. If *P* < .05 and *I*^2^ > 50%, it manifests that the study has significant statistical heterogeneity.

#### 2.3.6 Assessment of reporting bias

If there are >10 trials included in the study, the symmetry of the funnel plot will be drawn to detect reporting biases. The Egger test will be carried out for quantitative analysis.^[14]^

#### 2.3.7 Data synthesis

The RevMan 5.3 software (Version 5.3, Copenhagen: The Nordic Cochrane Center, The Cochrane Collaboration, 2014) provided by the Cochrane Collaboration will be applied to analyze data. Data will be analyzed with a fixed-effect model if no statistical heterogeneity was observed. In the presence of heterogeneity, a random-effect model will be used. Then, the possible causes of heterogeneity will be examined through the subgroup analysis or sensitivity analysis.

#### 2.3.8 Subgroup analysis

We will conduct subgroup analysis to detect possible heterogeneity based on different interventions, duration of treatment, and types of outcomes.

#### 2.3.9 Sensitivity analysis

To confirm the robustness of our findings, a sensitivity analysis will be conducted by ruling out studies of low quality. The meta-analysis will be performed again, and the results of these 2 meta-analyses will be compared. Thus, we will be able to assess the impact of low-quality studies on the overall results.

#### 2.3.10 Grading the quality of evidence

To assess the quality of evidence, the Grading of Recommendations Assessment, Development, and Evaluation (GRADE) will be used. The quality of evidence will be categorized into 4 levels: high, moderate, low, and very low quality.

#### 2.3.11 Ethics and dissemination

This systematic review will not require ethical approval because there are no data used in our study that are linked to individual patient data. The results will be disseminated only in a peer-reviewed publication.

## 3 Discussion

PMO is the most common generalized disease of the skeleton in women, as the lack of estrogen accelerates bone turnover with net bone loss. It is a common, asymptomatic postmenopausal disease that affects the quality of life of older women seriously. Thus, effective intervention should be conducted in the management of PMO. GSK, a classic Chinese patent medicine, has been clinically applied in the treatment of primary osteoporosis. In recent years, many clinical studies have suggested that GSK may have a good effect on PMO. However, there is no systematic review related to GSK for PMO. Therefore, we conduct this systematic review to further study the effectiveness of GSK in treating PMO. We hope the results of this study will provide more options for clinical treatment of the disease.

### 3.1 Amendments

If amendments are needed, we will update our protocol to include any changes in the whole process of research.

## Author contributions

**Data curation:** Guangwei Wang, Liwei Huo.

**Formal analysis:** Guangwei Wang, Liwei Huo.

**Methodology:** Guangwei Wang, Liwei Huo.

**Project administration:** Guangwei Wang.

**Resources:** Guangwei Wang, Liwei Huo, Guocai Chen.

**Software:** Guangwei Wang, Liwei Huo, Guocai Chen.

**Visualization:** Guangwei Wang, Liwei Huo, Guocai Chen.

**Writing – original draft:** Guangwei Wang.

**Writing – review & editing:** Guocai Chen, Huayong He.
